# A Case of Traumatic Tetanus in a Young Child, with Treatment and Recovery

**Published:** 1877-08

**Authors:** H. N. Burr

**Affiliations:** Wolworth, N. J.


A Case of Traumatic Tetanus in a Young Child, with Treatment and Recovery.—*By H. N. Burr, M.D., of Wolworth, N. J.* The following case is one of unusual interest to the profession, and for that reason I think it important that it should be published, for their benefit. The statement I think is entirely novel, and under unfavorable results might be severely criticised by some of the profession. I was called on the morning of October 8, 1873, to see Charles Laurence, a bright, active little boy, aged two years and nine months, who had been well up to this time, with the exception of occasional attacks of spasmodic croup, which had never continued but a short time. During the night of the seventh, he had been taken with a high fever and great restlessness; there was a jerking of the arms and legs, and also of the muscles of the body; and when asleep would start up with a jump and moan, as if in pain. When moved during the night it gave him pain; the head was hot, red, swollen, and tender to touch. A scar on the forehead had taken on a red and angry appearance, and radiating from it in different directions were deep red lines, which passed to the top of the forehead, and were lost in the hair; to account for the scar, I was told he had been bitten by the house dog eight days before, while he was teasing him; it bled profusely, but healed readily, leaving a scar that continued to look red up to this time. His condition when I saw him, on the morning of the 8th, was as follows: High fever, with the surface hot and dry; very nervous and irritable, and complains of being hurt when he is moved, as though the muscles were sore. He starts up with a cry every few minutes; the head is hot, with the part about the scar red and swollen. When he is moved he acts as if his back was stiff and sore. His breathing short and quick; the pulse full and very rapid, as much as 150, and even more, to the minute. There was a jerking of the muscles of the face, and of the arms and legs. When undisturbed for a few moments he would be quiet, and then start up with a jump that would raise him from his bed or his mother's lap. His eyes were bright, with the pupils about natural; he kept them closed unless he was disturbed. The temperature, I regret very much not to have taken, as in the study of the case I find it quite an important feature; some time during the night his mother had given him a dose of castor oil, which had not operated when I saw him. I ordered an injection of warm water to assist its action. I prescribed tincture of aconite root, in drop doses every hour, to reduce the pulse and subdue the dry hot surface, and

℞—Pulv. doveri., . . . . . . grs.iij
Potass. nitrat., . . . . . . grs.ij.

every three hours, with a cloth wet in cold water to his head.

On leaving him, I gave his parents to understannd that something serious would probably supervene. I saw him about eight in the morning. The injection was given, and his bowels moved freely, from its effects. At dinner he asked for soup, of which he ate some, but did not retain it but a short time, when it was vomited. Soon after this he became convulsed with a spasm of the arms and legs, and the whole body became stiff, and he sank into a deep sleep, from which he could not be aroused. He was, by his parents, put into a warm bath, which relaxed the muscles a little. His jaws were set and could not get him to swallow the least thing. When they found their efforts were of no avail, I was sent for, and saw him not far from 1 P. M. I found him lying in his crib, breathing with a slow and labored effort, with a rattle in his throat. In trying to get some liquid in his mouth I found the jaws were set, and after prying them open no effort would be made to swallow. The legs and arms were stiff, the hands were closed. Pulse full, strong, and bounding, the skin very hot but moist; the eyes were closed, but when opened the pupils were dilated very wide and sensitive to the light. His head was hot and the scar very red. Judging, from his breathing, and the rattle in his throat, that death was fast approaching, I concluded nothing could be done for him, and that he must die.

After a few moments reflection I thought of using cold water showered on his head. This was done to relieve the anxiety of his parents, and to have the appearance of doing something. I first gave him an injection of warm salt and warter, with turpentine, to divert the blood from the head. I then took him out of the crib, with his vital powers sinking, and commenced to pour cold water on his head. This I felt was my only hope, as he was unable to swallow the least thing, and there was the rattle which I had heard in several instsnces where patients had died from apoplexy. His head was held over a pail, and with a pitcher that would hold a quart I commenced pouring cold water, right from the well, in a small stream on the back of his head, letting it fall more than one foot. This was continued for three hours, with an occasional intermission of a few minutes at a time, when he sank into an easy slumber, and in a short time awoke, and was in his right mind. In connection with the cold water we used mustard to the back of the neck and soles of the feet. During the first half-hour there was but very little change in his condition, the water not seeming to be noticed. At the end of that time there was a change in his breathing, which became easier and more natural. The rattling had entirely subsided, which showed there was relief to the pneumogastric nerve. This change was apparent to all who observed him, but it was the only change until three-quarters of an hour had passed, when he moaned and opened his eyes for the first time. The eyes were dilated and extremely sensitive to the light. Soon after his breathing improved and he moaned, the rigidity of his muscles gave way, and we could get his mouth open sufficient to introduce anything into his mouth. The effort to swallow was terrible, and gave me the impression that he was choking. He would open his eyes, which were glaring and glassy, and raise himself almost from the pillow in his struggle to swallow, and I did not give more than half a teaspoonful at a time. I did not persist in giving him medicine by the mouth until I had used more water. If left for a few minutes without the water being poured on his head, he would sink away into the deep sleep, from which nothing would arouse him but the cold water poured on his head. As soon as I thought it safe to do so, I gave him a dose of morph. sulph., $\frac{1}{16}$ gr.; camph. pulv., 2 grs.; this I gave to get the effect of the morphine in contracting the pupil. At times he would moan, and cry out, and try to move, but took no notice of any one or anything, except the cold water, and not even that until it had been continued for some time without interruption, except long enough to replenish the pitcher. During the first hour there was not much improvement, except in the breathing and muscular relaxation, and during this time the water had been faithfully applied. A larger pitcher was now procured, and its contents could be poured in a small stream entirely on his head with no expression from him except to moan when the pitcher was nearly empty. The pitcher would be again filled and continued until he would come partially to his senses, when we could see by his actions that the intense cold was distressing him, when we would stop until he sank again to sleep. In an hour and a half I gave him another dose of the morphia and camphor, as the pupils were very largely dilated. It was about 4 P. M., when he sank into his natural sleep, and about five awoke, and knew his parents and all about him. I gave him $\frac{1}{16}$ grain morphia every four hours, to keep him quiet, and drop doses of the aconite as often as every hour, if it was required, to keep the fever down and also to keep him perspiring freely, and in case his skin became hot and dry to increase the amount. Bromide of potassium, five grains every two hours, to allay the jerking of the muscles of the arms and legs. He passed a quiet night, with some flashes of fever and jerking of the muscles.

9th. Saw him this morning, and found the pupils were not so largely dilated as yesterday; pulse not so strong nor so frequent, and not much fever. There is some jerking of the muscles, and starting up in his sleep, with a sharp irritable cry; also complains of its hurting his back when moved. The scalp swollen and red, with the angry look about the scar.

The urine is not high-colored. I had the scar painted with a solution of veratrum viride and glycerine. Ordered mustard to the back and feet, and continued the morphia and camphor, bromide potassium and aconite, renewing my instructions to keep him moist.

10th. This morning he is very much improved from yesterday, being bright and cheerful, with some of the nerve symptoms present, while the jerking was all gone. I continued all medicines except the bromide of potassium. I saw him again on the 11th, and could see no signs of the fearful disease about him, while the swelling and redness about the scalp had subsided and the scar had a more healthy look.

He continued well until the 30th, when his father called on me and said the boy was not so well as he had been, but was extremely nervous and irritable, with jerkings of the arms and legs, and jumping when he went to sleep. He was very much afraid the convulsions would return. The scar where he had been bitten was red and swollen, with some streaks running from it, and his head was hot. I gave him morphine and bromide of potassium, and ordered cold water to his head Under the use of these medicines his symptoms subsided, and in a day or two he was well. There were a number of times during the year when he would get very nervous and irritable, but at no time did he ever have spasms. The boy is now living, a bright, active little fellow. The dog was killed a few weeks after, but was not rabid; it having been growing cross toward the little boy for two or three weeks before he was bitten. Had the boy died, most people would have said it was from hydrophobia, but since he got well, that cannot be, and I must conclude I had a case of tetanus. I think now, but for the persistent use of the cold water he would have died, and I may attribute my success in this case to its use.—*Hospital Reports.*

